# Patterns of medicine use in the year prior to death by suicide: an Australian population-based case series study

**DOI:** 10.1016/j.eclinm.2024.102858

**Published:** 2024-10-03

**Authors:** Andrea L. Schaffer, Nicholas A. Buckley, Jennifer Schumann, Jessy S. Lim, Rose Cairns, Jacques Raubenheimer, Sallie-Anne Pearson, Kate Chitty

**Affiliations:** a Medicines Intelligence Research Program, School of Population Health, UNSW Sydney, Sydney, Australia; b Bennett Institute for Applied Data Science, Nuffield Department of Primary Care Health Sciences, University of Oxford, Oxford, UK; c Biomedical Informatics and Digital Health, Faculty of Medicine and Health, The University of Sydney, Sydney, Australia; d Victorian Institute of Forensic Medicine, Melbourne, Australia; e Department of Forensic Medicine, Monash University, Melbourne, Australia; f School of Pharmacy, Faculty of Medicine and Health, The University of Sydney, Sydney, Australia; g New South Wales Poisons Information Centre, The Children’s Hospital at Westmead, Westmead, Australia; h School of Population and Global Health, The University of Western Australia, Perth, Australia

**Keywords:** Suicide, Mental health, Drug utilisation, Pharmacotherapy, Australia

## Abstract

**Background:**

Prescribed medicines are commonly used to treat mental health conditions but are also often implicated in suicide death by poisoning. This was a descriptive study quantifying changes in dispensing and initiation of antidepressants, benzodiazepines, and antipsychotics in the year prior to death by suicide.

**Methods:**

In this Australian population-based case series, we used national coronial data linked with dispensing claims for all people ≥10 years who died by suicide (2013–2019). Our primary outcome was change in aggregate weekly medicine dispensing the year before death, quantified using piecewise linear regression stratified by cause of death (medicine poisoning vs other causes). Our secondary outcome was change in medicine initiation rates. This study was performed between June 2021 and July 2023.

**Findings:**

Our study included 14,207 people (24% female, median age 44  years). In the year prior to death, we observed higher rates of nervous system medicine use in people who died by medicine poisoning compared with those that did not: antidepressants (62.4% vs 42.9%), benzodiazepines (51.4% vs 29.0%), antipsychotics (25.6% vs 17.1%), opioids (43.8% vs 23.4%). For benzodiazepines, among people who died by medicine poisoning the slope (rate of increase) changed from 0.18 (95% CI −0.01, 0.37) to 4.12 (95% CI 0.98, 7.26) dispensings per 1000 people per week at 8 weeks prior to death. Among people who died of other causes, the slope changed from 0.18 (95% CI 0.14, 0.22) to 2.41 (95% CI 1.90, 2.91) also at 8 weeks prior to death. For antidepressants, among people who died of medicine poisoning we observed no change in the slope. Among people who died of other causes, the slope increased from 0.18 (95% CI 0.09, 0.28) to 1.68 (95% CI 1.20, 2.15) at 14 weeks prior to death.

**Interpretation:**

Dispensing of antidepressants and benzodiazepines increased more rapidly closer to date of death, regardless of medicine involvement in death. This suggests these changes may reflect worsening symptoms or increased help seeking and that the method of death by suicide may be due to greater means access. However, findings need to be interpreted with caution as our analyses were performed on aggregate data and may not reflect person-level changes.

**Funding:**

This study is funded by a grant from the Australian National Health and Medical Research Council (NHMRC) and the Translational Australian Clinical Toxicology Research (TACT) Group.

**Research in context**

**Evidence before this study**

We searched PubMed for publications between 1 January 2000 and 31 January 2024 using the following search terms: “suicide” AND (“medicine” OR “psychotropic” OR “antidepressant” OR “benzodiazepine” OR “antipsychotic”) AND (“prescribing” OR “dispensing” OR “prescription”) AND (“trajectory” OR “pattern” OR “changes”). We also searched the reference lists of relevant articles. We included observational research studies, excluding conference abstracts and editorials, quantifying changing patterns of medicine use prior to death by suicide or suicide attempt.

We identified four studies. A 2019 Norwegian study of 594 people who died by suicide found that 74% of females and 63% of males were prescribed psychotropic medicines in the 12 months prior to death; the corresponding percentages in the 30 days prior to death were 51% and 36%. A 2021 Swedish study of 62,442 people attempting suicide found approximately 11% of Swedish-born people and 6% of refugees had increasing antidepressant use in the year prior to death, 10% and 7% had high constant use, 19% and 23% had medium constant use and 60% and 65% had low constant use.

A 2018 study of 5070 US adults who presented to the emergency department for suicidal behaviour found a spike in antidepressant prescribing the month before presentation. Lastly, a 2023 Australian study of 3895 people who died by suicide identified 5 trajectories of health service utilisation in the year prior to death, each group with distinct levels and patterns of mental health and physical health medicine dispensing. High use of mental health medicines in the year before death was observed for three of the five trajectories.

**Added value of this study**

While many studies have quantified changes in health service utilisation (e.g., hospitalisations, emergency attendances, primary care contacts, mental health contacts) prior to death by suicide, very few have quantified changing patterns of medicine dispensing or prescribing. Our study comprises one of the largest cohorts of people who died by suicide (n = 14,207) and describes changes in patterns of medicine dispensing before death. A strength of this study is that medicine dispensing data were linked with toxicology data, which allowed us to distinguish between people who died by medicine poisoning and those who did not, and to determine whether the dispensed medicine was subsequently detected at autopsy. Therefore, we were able to provide data on whether medicine dispensing patterns differed depending on cause of death (medicine poisoning or other).

**Implications of all the available evidence**

While the ecological nature of our analysis limits what can be inferred, our findings suggest that the observed increases in medicine dispensing prior to death by suicide are likely to represent worsening or changing mental health symptoms, or increased help-seeking for existing conditions, rather than obtaining medicines with the intent of self-harm. Medicine prescribing presents an opportunity for health professionals to directly engage with patients to identify people with worsening symptoms at risk of self-harm or potential suicidal behaviour. We cannot rule out other explanations, such as medicines obtained with the intent of self-harm even if not resulting in death, and it cannot be determined whether medicine initiation increases suicidality.

## Introduction

Worldwide, one in 100 deaths are a result of suicide, with a global average of 9 suicide deaths per 100,000 people.^1^ The impact of suicide is widespread; based on a 2020–2022 survey of 16,000 Australians aged 16–85 years, one in six have had serious suicidal thoughts, nearly 5% have attempted suicide, and more than one third (36%) were close to someone who attempted or died by suicide in their lifetime.^2^ While there are many contributing factors to suicide and self-harm, a leading risk factor for death by suicide is mental illness.^3^

Several studies investigated the health care utilisation of suicide decedents prior to death. Point estimates are variable across countries and methodologies, but a consistent finding is that while contact with primary healthcare or hospitals in the year prior to death is common, fewer people who died by suicide have mental health contacts.^4^^,^^5^ However, this varies by sex, with females much more likely than males to have used mental health services, to have had a mental health hospitalisation, or to have been dispensed a mental health medicine.^2^^,^^4^^,^^5^ Psychotropic medicines are a standard treatment for depression and other mental health problems, yet a substantial proportion of Australians who die by suicide will not be using them at the time of death, as confirmed by toxicology.^6^ Nonetheless, the relationship between medicine use and suicide is complex. Poisoning is the cause of death for approximately one in four deaths by suicide with most of these deaths involving medicines.^7^ Some antidepressants have been associated with an increased risk of suicidal behaviour during the initiation period. However, the true relationship is unclear as many of the studies have designs that do not address common biases in observational studies, in particular confounding by indication.^8^ While means restriction (i.e., reducing the risk of suicide by limiting access to lethal means) is a key element of suicide prevention,^9^^,^^10^ pharmacotherapy with psychotropic medicines is often necessary to treat mental health symptoms that may precipitate suicide.

Understanding how medicine use changes preceding prior to death by suicide may provide insight into how mental health symptoms are changing and identify potential points of intervention for suicide prevention. Given the dual role of medicines in both preventing suicide and as a potential means of death, it is informative to disaggregate data by cause of death, specifically whether it involved medicine poisoning to help clarify the roles of medicines in choice of method of death suicide. Therefore, among all people ≥10 years of age who died by suicide from July 2013 through October 2019 in Australia, we quantified the: 1) prevalence and incidence of medicine dispensing; and 2) changes in dispensing patterns in the 12 months prior to death by suicide. We compared these outcomes among people who died by medicine poisoning with people who died by other causes, for both psychotropic and non-psychotropic medicines.

## Methods

### Study population

In this Australian population-based case series, we used data from the Australian Suicide Prevention using Health Linked Data (ASHLi) cohort which is described in detail elsewhere.^11^ This is a nation-wide, population-based case series of all people who died by suicide in Australia identified through the National Coronial Information System (NCIS) from 1 July 2013 to 10 October 2019. The NCIS captures all deaths notified to a coroner in Australia and New Zealand, including those that are of intentional, accidental, or undetermined intent.^12^ An overview of the coronial system in Australia is provided in Phillips et al.^13^ Due to small numbers and the sensitive nature of death by suicide in children and the difficulty in determining intent, we restricted our population to people aged ≥10 years.^14^ This study was performed between June 2021 and July 2023.

### Data sources

The NCIS data captures demographics, autopsy and toxicology findings and details surrounding the death (including cause of death). Our study population identified via the NCIS was linked with the Pharmaceutical Benefits Scheme (PBS) and Repatriation PBS dispensing claims. The Pharmaceutical Benefits Scheme (PBS) is part of Australia’s universal healthcare system and subsidises medicines to Australian citizens and eligible recipients. All Australian citizens and eligible residents receive subsidised access to prescribed medicines through the PBS, while eligible veterans and their dependents receive access to additional items through the RPBS. These data capture medicines dispensed in the community and private hospitals across Australia. Private dispensings, over-the-counter medicines, and dispensings to public hospital inpatients are not captured in the PBS collection.^15^ Private dispensings refer to medicines that are prescribed outside the subsidy criteria (which may be due to off-label use) or are priced outside the recommended pricing range.

### Cause of death

We performed analyses both overall and stratified according to whether the cause of death was medicine poisoning or not. In NCIS data, the coroner records cause of death in a free-text field and the mechanism and object of injury are recorded in coded categorical fields. ICD-10 codes are externally supplied by the Australian Bureau of Statistics. We searched each data field to determine whether poisoning was involved in the cause of death and the type of poison(s) involved. “Medicine poisoning related” deaths were classified based on the coroner’s finding and included all deaths where one or more prescription-only or over-the-counter medicine(s) were determined to contribute to the death to any extent, even if not the primary cause (e.g., *a death by hanging with toxic levels of benzodiazepines* was considered medicine poisoning related). For stratified analyses, people for whom medicine involvement could not definitively be determined were excluded.

### Medicine use measures

First, we quantified overall dispensing for all prescription medicines as a measure of overall health and interaction with the healthcare system. Medicines were classified using the World Health Organisation (WHO) Anatomic Therapeutic Chemical (ATC) classification. The primary medicines of interest were nervous system medicines (WHO ATC category N).

We focussed primarily on medicines commonly used to treat mental health symptoms and/or often involved in poisonings, specifically: antidepressants (N06A), benzodiazepines (N05B, N05C), antipsychotics (N05A, excluding lithium) and opioids (N02A). We also performed an analysis of all N-class medicines combined; in addition to those mentioned above, this also included medicines such an antiepileptics and psychostimulants. A full list of included medicines which were dispensed at least once in our data is in Supplementary Table S1.

Additionally, we identified the three most commonly dispensed classes of non-nervous system medicines in our study population to establish whether observed patterns were specific to psychotropic medicines. These were: angiotensin converting enzyme inhibitors and angiotensin receptor blockers (ACEI/ARBs) (WHO ATC code C09); medicines to treat gastro-oesophageal reflux disease (GORD) (WHO ATC code A02B); and antibiotics (WHO ATC code J01).

First, we quantified prevalent dispensing of medicines, both over the entire 12-month period, and by week prior to death. The denominator for calculation of rates and percentages was the entire population. Second, we identified initiation in the last 6 months (180 days) prior to death and used two definitions of initiation. The primary definition (“class-level initiation”) referred to initiation of any medicine in a particular class (i.e., antidepressants, benzodiazepines, antipsychotics, opioids). A person was considered to have initiated a specific medicine class if they had no dispensing of that class for at least 180 days prior to their first observed dispensing. When calculating the rates of initiation, the denominator was the number of persons with no recent evidence of use of that medicine class (“treatment-naïve”), defined as no dispensing for a medicine in that class for at least 180 days. This denominator was used because in this definition, only people naïve to that medicine class could initiate.

For the secondary definition (“medicine-level initiation”), a person was considered to have initiated if they had a first observed dispensing for a specific medicine (e.g., fluoxetine, diazepam) in the 180 days prior to death. For this definition, a person may have been dispensed a different medicine in that class previously. Here, the denominator was everyone in the cohort, as everyone could initiate a new medicine even if they were already taken one in the same class and this could represent an add-on or switch to a different specific medicine within a given medicine class (e.g., from paroxetine to fluoxetine which are both SSRIs).

### Statistics

To assess changes in medicine use in the year prior to death, we performed linear regression with time as the independent variable. For overall dispensing, the dependent variable was the number of dispensings per 1000 people per week over the 52 weeks prior to death. For initiation, we focussed on antidepressants and benzodiazepines only, due to small initiation counts for other medicine classes. Here, the dependent variable was the number of people initiating an antidepressant or benzodiazepine per 1000 treatment-naïve people per two-week period for the 26 weeks prior to death. Two-week periods were used instead of weeks due to small counts.

We identified structural changes in the relationship between time and our outcomes of interest, using the *segmented* R package which performs a piecewise linear regression and selects the position of breakpoints based on a sequential hypothesis testing procedure.^16^ A breakpoint is when the slope (rate of change) of the regression line changes. The number of breakpoints was chosen based on the model with the lowest BIC. This was performed separately for each outcome and medicine class of interest, as well as stratified by cause of death (medicine poisoning vs other causes). As the primary objective of our study is descriptive (not causal), we have not adjusted for age or sex in our primary analysis^17^^,^^18^; however, we present some sex and age disaggregated data for reference.

As a sensitivity analysis, we repeated the analysis of change in dispensing for antidepressants, benzodiazepines, antipsychotics and opioids restricted to people for whom these medicines were detected during toxicological testing during autopsy. For opioids, this included both prescribed opioids and illicit opiates (e.g., heroin) as these could not be distinguished.^6^

### Ethics

This study was approved by the following ethics committees: Department of Justice and Community Safety Justice Human Research Ethics Committee (CF/17/23,250), Western Australian Coronial Ethics Committee (EC14/18); Australian Institute of Health and Welfare (AIHW; EO2017/4/366), and the New South Wales (NSW) Population & Health Services Research Ethics Committee (2017/HRE1204). Individual consent was waived.

### Role of the funding source

The funding organisations played no part in the design of the study; nor in the preparation, review, or approval of the manuscript.

## Results

Our study population included 14,207 people ≥10 years who died by suicide. The median age was 44 years (interquartile range [IQR] 31–57) and 75.7% were male (Table 1). Most deaths did not involve medicine poisoning (85.4%, n = 12,137); 8.5% of cases (n = 1210) involved medicine poisoning. For the remaining 6.1% (n = 860) the involvement of medicines could not be determined, as such they were excluded from all analyses stratified by medicine involvement (but included in overall analyses). People dying by medicine poisoning were older (median age = 54 years, IQR = 41–66) than decedents who died of other causes (mean age = 43 years, IQR = 30–56), and were more likely to be female (47.3% vs 19.8%).

**Table 1:** Characteristics of people ≥10 years who died by suicide, Jul 2013–Oct 2019.

		Cause of death^a^	
	All decedents	Medicine poisoning	Other causes (not medicine poisoning)
	N (%)	N (%)	N (%)
Total	14,207 (100.0)	1210 (100.0)	12,137 (100.0)
Year of death^b^			
2013–2014	3908 (27.5)	374 (30.9)	3303 (27.2)
2015–2016	5428 (38.2)	475 (39.3)	4633 (38.2)
2017–2019	4871 (34.3)	361 (29.8)	4201 (34.6)
Male	10,758 (75.7)	645 (52.7)	9734 (80.2)
Female	3449 (24.3)	578 (47.3)	2403 (19.8)
Age			
10–24 y	1920 (13.5)	61 (5.0)	1817 (15.0)
25–44 y	5305 (37.3)	315 (26.0)	4741 (39.1)
45–64 y	4744 (33.4)	501 (41.4)	3848 (31.7)
65+ y	2238 (15.8)	333 (27.5)	1731 (14.3)
Age, median (IQR)	44 (31–57)	54 (41–66)	43 (30–56)
Cause of death			
Medicine poisoning	1210 (8.5)	1210 (100.0)	–
Other causes (not medicine poisoning)	12,137 (85.4)	–	12,137 (100.0)
Medicine involvement could not be determined	860 (6.1)	–	–
Medicine detected at death			
Antidepressants	4607 (32.4)	589 (48.7)	3435 (28.3)
Benzodiazepines	4162 (29.3)	624 (51.6)	3935 (24.2)
Antipsychotics	1753 (12.3)	259 (21.4)	1211 (10.0)
Opioids^c^	2572 (18.1)	533 (44.1)	1508 (12.4)
No toxicology data available	665 (4.7)	592 (4.9)	47 (3.9)

a Excludes n = 860 where involvement of medicines in poisoning could not be definitively determined.

b Cases included from 1 July 2013 to 10 October 2019.

c Includes both prescribed and illicit opiates.

### Prevalence and incidence of medicine use in the year prior to death by suicide

Nearly one in five people (n = 2603; 18.3%) in the full cohort did not have any PBS-listed medicine dispensed in the year prior to death (Table 2). This was more common in people where medicine poisoning did not contribute to death than for those where it did (n = 2497 [20.7%] vs n = 70 [5.8%]).

**Table 2:** Medicine dispensing characteristics in year prior to death by suicide in people ≥10 years, 2013–2019.

Patterns of medicine use in year prior to death	All decedents	Cause of death^b^	
		Medicine poisoning	Other causes (not medicine poisoning)
Total, n (%)	14,207 (100.0)	1210 (100.0)	12,137 (100.0)
≥1 dispensing for any medicine, n (%)	11,604 (81.7)	1140 (94.2)	9640 (79.3)
No. total dispensings, median (IQR)	10 (2–32)	34 (12–66)	7 (1–24)
No. unique medicines dispensed, median (IQR)			
Overall	4 (1–7)	7 (4–12)	3 (1–6)
Nervous system medicines^a^	1 (0–3)	3 (1–5)	1 (0–3)
Nervous system medicines^a^ dispensed, n (%)			
Antidepressants	6638 (46.7)	755 (62.4)	5204 (42.9)
Benzodiazepines	4695 (33.0)	622 (51.4)	3517 (29.0)
Antipsychotics	2689 (18.9)	310 (25.6)	2076 (17.1)
Opioids	3844 (27.1)	530 (43.8)	2840 (23.4)
Non-nervous system medicines dispensed, n (%)			
Antibiotics	6219 (43.8)	657 (54.3)	5037 (41.5)
Gastro-oesophageal reflux disease	2703 (19.0)	389 (32.1)	1966 (16.2)
ACEI/ARBs	2225 (15.7)	286 (23.6)	1728 (14.2)

IQR = interquartile range.

a Refers to N-class medicines as defined by the WHO Anatomic Therapeutic Chemical (ATC) Classification.

b Excludes n = 860 where involvement of medicines in poisoning could not be definitively determined.

People who died by medicine poisoning had a much greater number of dispensings in the year prior to death (median = 34) compared with those who died of other causes (median = 7), and a greater number of unique medicines dispensed (median = 7 vs median = 3). The most commonly dispensed nervous system medicines in the year prior to death were antidepressants, dispensed to 6638 people (46.7%), followed by benzodiazepines (n = 4695; 33.0%), opioids (n = 3844; 27.1%), and antipsychotics (n = 2689; 18.9%). The most common non-nervous system medicines were antibiotics (n = 6219; 43.8%), medicines to treat gastro-oesophageal reflux disease (GORD) (n = 2703; 19.0%), and ACEI/ARBs (n = 2225; 15.7%). Dispensing of all medicine classes was higher in people who died by medicine poisoning (Table 2; Supplementary Table S2). This is consistent with higher rates of medicine dispensing in females and older people (Supplementary Fig. S1).

Initiation of a new medicine class among treatment-naïve (no dispensing in 180 days) was highest for antidepressants (19.1%, 1785/9354), followed by benzodiazepines (15.5%, 1741/11,253), antipsychotics (6.5%, 798/12,316) and opioids (10.7%, 1235/11,598) (Table 3). Initiation rates were slightly higher in people who died by medicine poisoning, especially for benzodiazepines and opioids. Higher rates in people who died by medicine poisoning were also observed for non-nervous system medicines, especially antibiotics. Similar patterns were observed for the 3 months prior to death (Supplementary Table S3).

**Table 3:** Initiation rates of medicines by cause of death in the 6 months prior to death, 2013–19.

Class-level initiation among treatment naïve	Overall (n = 14,207)		Cause of death^a^			
			Medicine poisoning (n = 1210)		Other causes (n = 12,137)	
	No. initiators/No. treatment-naïve	%	No. initiators/No. treatment-naïve	%	No. initiators/No. treatment-naïve	%
Nervous system medicines						
Antidepressants	1785/9354	19.1	123/578	21.3	1583/8516	18.6
Benzodiazepines	1741/11,253	15.5	168/756	22.2	1445/10,065	14.4
Antipsychotics	798/12,316	6.5	77/977	7.9	657/10,718	6.1
Opioids	1235/11,598	10.7	116/796	14.6	1022/10,319	9.9
Non-nervous system medicines						
ACEI/ARBs	210/12,192	1.7	28/952	2.9	161/10,570	1.5
GORD medicines	577/12,081	4.8	66/887	7.4	449/10,620	4.2
Antibiotics	2107/10,095	20.9	190/743	25.6	1772/8872	20.0
**Medicine-level initiation among full population**	**No. initiators/Total population**	**%**	**No. initiators/Total population**	**%**	**No. initiators/Total population**	**%**
Nervous system medicines						
Antidepressants	2874/14,207	20.2	254/1210	21.0	2380/12,137	19.6
Benzodiazepines	2206/14,207	15.5	231/1210	19.1	1773/12,137	14.6
Antipsychotics	1153/14,207	8.1	118/1210	9.8	926/12,137	7.6
Opioids	1733/14,207	12.2	211/1210	17.4	1320/12,137	10.9
Non-nervous system medicines						
ACEI/ARBs	374/14,207	2.6	43/1210	3.6	288/12,137	2.4
GORD medicines	778/14,207	5.5	94/1210	7.8	590/12,137	4.9
Antibiotics	3517/14,207	24.8	391/1210	32.3	2806/12,137	23.1

Class initiation refers to initiation of any medicine within the class, while medicine initiation refers to initiation of an individual medicine. Treatment-naïve are people with no dispensings in the past 6 months.

ACEI/ARBs = Angiotensin-converting enzyme inhibitors/angiotensin-receptor blockers; GORD = Gastro-oesophageal reflux disease.

a Excludes n = 860 where involvement of medicines in poisoning could not be definitively determined.

### Changes in dispensing patterns in the year prior to death

**Fig. 1:**
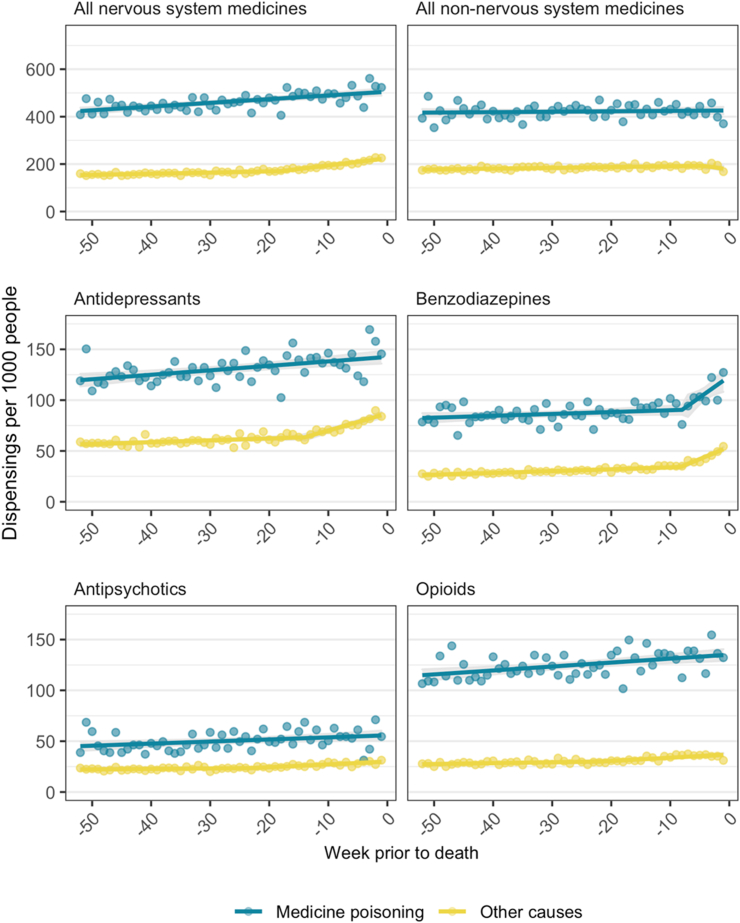
Medicine dispensing by week prior to death, stratified by cause of death (2013–2019). Points represent observed values, the solid line represents predicted values, shaded area represents 95% confidence interval for predicted values.

People who died by medicine poisoning had a consistently higher rate of dispensing of all medicines throughout the 12 months prior to death (Fig. 1). Higher rates of medicines dispensing were observed for people who died by medicine poisoning even after stratifying by age and sex to account for differences in age/sex distribution (Supplementary Fig. S1). Weekly dispensing of nervous system medicines gradually increased over time for people who died by medicine poisoning, whereas for people who died of other causes, dispensing rates were relatively stable with a breakpoint observed at 18 weeks prior to death, after which dispensing started to increase (Supplementary Table S4). We observed little change in dispensing over time for non-nervous system medicines in both groups.

For antidepressants, weekly dispensing rates remained high throughout the year for people who died by medicine poisoning and were increasing slightly over time. In the group who died of other causes, dispensing rates were lower with a breakpoint observed at 14 weeks prior to death after which dispensing increased. For benzodiazepines, despite different baseline dispensing rates a similar pattern was observed for both groups, with dispensing increasing rapidly at 8 weeks prior to death. Only very small changes were observed for antipsychotics and opioids (Fig. 1, Supplementary Table S4). We observed decreases in dispensing antibiotics and ACEI/ARBs prior to death, but no change in dispensing for GORD medicines (Supplementary Fig. S2).

Among people for whom the medicine class of interest was detected in post-mortem toxicological analysis, the weekly dispensing rate was higher than in the overall decedent population, regardless of cause of death (Supplementary Fg. S3 and Table S5). Compared with the primary analysis, the timing of the breakpoints was similar, however the rate of increase in the second segment was much steeper among people for whom the medicine was detected at death, especially for people who died of other causes (not medicine poisoning).

### Change in initiation in the 6 months prior to death

**Fig. 2:**
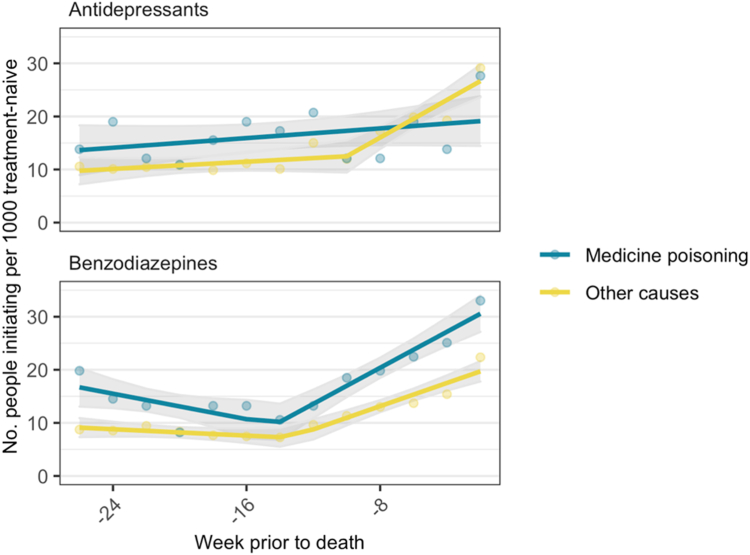
Medicine class initiation by two-week periods prior to death, stratified by cause of death (2013–2019). Points represent observed values, line represents predicted values, shaded area represents 95% confidence interval for predicted values.

Initiation of antidepressants and benzodiazepines peaked in the two weeks prior to death (Fig. 2). For people who died of other causes, there was a breakpoint at 10 and 14 weeks prior to death for antidepressants and benzodiazepines respectively. For people who died by medicine poisoning, a breakpoint was observed for benzodiazepines only at 14 weeks prior to death (Supplementary Table S6).

## Discussion

We have quantified differences in medicine use prior to death, stratified by cause of death and demonstrated that people dying by medicine poisoning have higher rates of dispensing of all medicines, both medicines commonly used in poisonings (e.g., antidepressants, benzodiazepines), and those to treat non-mental health conditions as well. We also found that while dispensing and initiation of many psychotropic medicines started to increase in the months leading up to death, this was more commonly observed among people who died of causes other than medicine poisoning.

We observed relatively modest changes in dispensing of most psychotropic medicines in the year prior to death. However, we saw a dramatic increase in the dispensing of benzodiazepines a few months prior to death, regardless of cause of death and whether benzodiazepines were detected by toxicological analysis. Benzodiazepines are primarily recommended for treatment of insomnia and severe anxiety disorders, but not recommended for treatment of depression.^19^ Increased dispensing of benzodiazepines prior to death by suicide could reflect increased anxiety, agitation and sleeplessness that occurs during times of distress. That increased dispensing of benzodiazepines occurred in those that did not die from medicine poisoning provides some more context to the finding that they are one of the most commonly detected medicines in suicidal overdoses and deaths by suicide both internationally^20^ and in Australia,^6^ despite being less lethal than many other drugs commonly detected in overdose.^20^ However, benzodiazepines are intoxicating and may lead to increased impulsivity.^21^ They are also more likely to lead to death when combined with other sedative medicines,^22^ and are common in polydrug overdoses.^7^^,^^23^

In deaths by suicide, benzodiazepines are more commonly observed in combination with antidepressants rather than alone.^7^ Antidepressants have a delayed onset of action but can cause increased agitation and insomnia initially, and benzodiazepines may be used to treat these symptoms despite the risks.^19^ We also observed increases in initiation of benzodiazepines (regardless of cause of death) and antidepressants (for people who did not die by medicine poisoning only). While some psychotropic medicines have been associated with an increase in suicidal ideation,^24^^,^^25^ this cannot be distinguished from treatment provided for increasing mental health symptoms in this study. The purpose of this study was not to determine the risk of death associated with certain medicine use, and this is not possible without a non-decedent control group.

Of note is that opioid dispensing was very common, dispensed to over one quarter of people in our cohort. Opioids are also commonly involved in poisoning suicides^26^ and are relatively more toxic than other medicines^20^ especially when used in combination with other sedatives. Opioids are commonly prescribed for chronic non-cancer pain (despite a lack of evidence for this indication^27^) which often co-occurs with depression and sleep problems, and chronic pain is itself a risk factor for suicide and suicidal ideation.^28^^,^^29^ Conditions commonly treated with opioids, such as cancer, also increase the risk of suicide.^30^^,^^31^ At the same time, stopping opioid therapy has also been associated with an increased risk of suicide.^32^

Similar dispensing patterns were observed regardless of cause of death and the largest increases in dispensing in the months leading to death were for people who did not die by medicine poisoning. This was observed even when restricted to people for whom these medicines were identified in toxicology. One potential explanation is that these increases in dispensing reflect worsening mental health symptoms, which may either lead to people refilling their prescriptions more frequently, or an increase in help-seeking behaviour resulting in greater interaction with healthcare providers, who may in turn prescribe psychotropic medicines to treat symptoms. In our previous work looking at changes to healthcare interactions among people who died by suicide in New South Wales only (n = 3895),^33^ healthcare contact overall was low in the year prior to death from suicide and increased healthcare access in the few months prior to death was only observed among a minority of individuals. That said, if our findings were the result of an increase in healthcare interactions alone, we would have also expected to see an increase in dispensing of our control medicines, which was not apparent in the current study. Regardless, the relationship between increasing mental health symptoms, healthcare interactions, and dispensing patterns is difficult to fully disentangle.

It is also important to note that these changes were observed at the aggregate dispensing level, and we cannot say whether these increases would also occur at the person-level as it may be different people dispensed medicines in each week. While the ecological nature of our analysis limits what can be inferred, our findings suggest that medicine poisoning involvement in death by suicide may be due to greater access to lethal means. We cannot rule out other explanations, such as medicines obtained with the intent of self-harm even if not resulting in death, and it cannot be determined whether medicine initiation increases suicidality. Regardless, caution and monitoring when prescribing toxic medicines for patients at risk of suicide are key to prevention, and approaches such as limiting the pack size has been used by some jurisdictions to reduce self-poisonings, such as with paracetamol in the UK.^34^ Medicine prescribing also presents an opportunity for health professionals to directly engage with patients to identify worsening symptoms and people at risk of self-harm or potential suicidal behaviour.

The strength of this study is the population-based sample of people who died by suicide, the detailed coronial information allowing us to identify cause of death, and the inclusion of non-mental health medicines as controls. We also included adolescents and children ≥10 years, an understudied population in suicide research. However, we did not have data on non-fatal self-harm and suicidal behaviour, and some of our results may have differed had that been included. In particular, non-fatal self-harm is much more common in females,^2^ and is often associated with different suicide methods, with drug overdose being relatively more common in non-fatal suicidal behaviour compared with other methods. Mental health diagnoses are also not captured in our data, and therefore we could not ascertain whether people were using these medicines to treat mental health symptoms, or for other reasons. We focussed on changes in dispensing rates at the population-level using stratified analyses to identify how patterns varied by method of death and specific medicines; however, these changes may not necessarily reflect changes at the person-level as they may be the result of the ecological fallacy and should be taken with caution. We also don’t have information on medicines not prescribed and dispensed through the PBS, including off-label use, or obtained illicitly. However, in Australia the majority of medicines are subsidised and thus would be captured.^15^ We also could not examine changes in dispensing of less frequently dispensed medicines, such as mood stabilisers, or specific classes of antidepressants (e.g., TCAs) which are known to have increased toxicity in overdose.^22^ Finally, our study focussed on dispensing of medicines only, not the underlying healthcare appointment where the medicine was prescribed. Further work to delineate the relationship between prescribing and dispensing will improve the ability to provide clear recommendations to prescribers.

## Contributors

Conceptualization: ALS, KC; Data curation: ALS, JR, KC; Formal analysis: ALS; Funding acquisition: KC, ALS, JS, RC, NAB, SAP; Investigation: ALS; Methodology: ALS, KC, NAB; Project administration: ALS, KC; Resources: KC, NAB; Software: ALS, JSL, JR; Supervision: ALS, KC; Validation: ALS, JSL, JR, KC; Visualisation: ALS; Writing - original draft: ALS; Writing - review & editing: All authors. ALS, JSL, and JR verified the underlying data. All authors gave final approval of the version to be published and agree to be accountable for all aspects of the work. All authors confirm that they had full access to all the data in the study and accept responsibility to submit for publication.

## Data sharing statement

Only approved personnel are permitted to access the data. Researchers interested in collaborations or further information are invited to contact NAB at nicholas.buckley@sydney.edu.au.

## Declaration of interests

RC is named on an untied educational grant from Reckitt to study over-the-counter medicines poisoning. RC also received an honorarium and travel costs from Reckitt to present educational information on poisoning prevention at a conference, and an honorarium from the Pharmacy Guild of Australia to present at a conference. These are unrelated to the present work. JSL received support from the Australian Government Research Training Program scholarship.
