# Supplementary material for: Patterns of medicine use in the year prior to death by suicide: an Australian population-based case series study
Source: eClinicalMedicine. 2024 Oct 3;77:102858. doi: 10.1016/j.eclinm.2024.102858 (PMC11474424; doi:10.1016/j.eclinm.2024.102858)
Supplement: Supplementary Figs. S1–S3 and Tables S1–S6 [file mmc1.pdf]

SUPPLEMENTARY FILES

Supplementary Table 1. List of medicines of interest that are dispensed at least once in ASHLi cohort

Medicine class	List of medicines appearing in data
**Antidepressants (N06A)**	*Selective serotonin reuptake inhibitors (SSRIs)*<br>Citalopram, Paroxetine<br>Escitalopram, Sertraline<br>Fluoxetine<br><br>*Serotonin and norepinephrine reuptake inhibitors (SNRIs)*<br>Desvenlafaxine, Venlafaxine<br>Duloxetine<br><br>*Tricyclic antidepressants (TCAs)*<br>Amitriptyline, Doxepin<br>Clomipramine, Imipramine<br>Dosulepin, Nortriptyline<br><br>*Other*<br>Mianserin, Phenelzine<br>Mirtazapine, Reboxetine<br>Moclobemide, Tranylcypromine
**Benzodiazepines (N05B, N05C)**	Alprazolam, Oxazepam<br>Diazepam, Temazepam<br>Nitrazepam, Zopiclone^
**Antipsychotics (N05A)**	Amisulpride, Lurasidone<br>Aripiprazole, Quetiapine<br>Asenapine, Olanzapine<br>Brexpiprazole, Paliperidone<br>Chlorpromazine, Periciazine<br>Clozapine, Risperidone<br>Flupenthixol decanoate, Trifluoperazine<br>Fluphenazine decanoate, Ziprasidone<br>Haloperidol, Zuclopenthixol decanoate
**Opioids (N02A)**	Buprenorphine[#], Morphine<br>Codeine, Oxycodone/ oxycodone + naloxone<br>Fentanyl, Paracetamol + codeine<br>Hydromorphone, Tapentadol<br>Methadone[#], Tramadol
**Other N-class medicines**	
**Other analgesics (N02)**	Paracetamol, cyproheptadine, eletriptan, naratriptan, pizotifen, rizatriptan, sumatriptan, zolmitriptan
**Antiepileptics (N03)**	Carbamazepine, clonazepam, gabapentin, lacosamide, lamotrigine, levetiracetam, oxcarbazepine, phenobarbital, phenytoin, pregabalin, primidone, tiagabine, topiramate, valproate, vigabatrin, zonisamide
**Antiparkinson (N04)**	Benzatropine, biperiden, trihexyphenidyl, amantadine, cabergoline, entacapone, levodopa + benserazide, levodopa + carbidopa,

	levodopa + carbidopa + entacapone, pramipexole, rasagiline, rotigotine, selegiline
**Psychostimulants (N06B)**	Atomoxetine, dexamfetamine, lisdexamfetamine, methylphenidate, modafinil
**Antidementia (N06D)**	donepezil, galantamine, memantine, rivastigmine
**Addictive disorders (N07B)**	Acamprosate, bupropion*, naltrexone, nicotine, varenicline
**Other nervous system**	Lithium, bethanechol, pyridostigmine, riluzole, tetrabenazine,

^Zopiclone is only subsidised for Australian Department of Veterans' Affairs clients

*Buproprion is only indicated on the PBS for treatment of nicotine addiction and thus is not included in the antidepressant category

#Methadone and buprenorphine for pain only, not opiate agonist treatment for dependence

Supplementary Table 2. Dispensing patterns in year prior to death by suicide in people ≥10 years (2013-2019). IQR = interquartile range.

Patterns of medicine use in year prior to death	Overall (n=14207)	Cause of death* Medicine poisoning (n=1210)	Cause of death* Other causes (n=12137)
**All medicines**			
≥1 dispensing, n (%)	11604 (81.7)	1140 (94.2)	9630 (79.3)
No. dispensings among people with ≥1 dispensing, median (IQR)	15 (5-39)	38 (14-68)	13 (4-31)
**NERVOUS SYSTEM MEDICINES**			
**All nervous system medicines**			
≥1 dispensing, n (%)	9539 (67.1)	1028 (85.0)	7711 (63.5)
No. dispensings among people with ≥1 dispensing, median (IQR)	3 (1-4)	4 (2-6)	2 (1-4)
**Benzodiazepines**			
≥1 dispensing, n (%)	4695 (33.0)	622 (51.4)	3517 (29.0)
No. dispensings among people with ≥1 dispensing, median (IQR)	3 (1-8)	5 (2-12)	2 (10-6)
**Antidepressants**			
≥1 dispensing, n (%)	6638 (46.7)	755 (62.4)	5204 (42.9)
No. dispensings among people with ≥1 dispensing, median (IQR)	7 (2-13)	10 (5-14)	6 (2-12)
**Antipsychotics**			
≥1 dispensing, n (%)	2689 (18.9)	310 (25.6)	2076 (17.1)
No. dispensings among people with ≥1 dispensing, median (IQR)	5 (2-12)	7 (3-14)	5 (2-11)
**Opioids**			
≥1 dispensing, n (%)	3844 (27.1)	530 (43.8)	2840 (23.4)
No. dispensings among people with ≥1 dispensing, median (IQR)	3 (1-11)	8 (2-20)	2 (1-7)

NON-NERVOUS SYSTEM MEDICINES			
**ACEI/ARBs**			
≥1 dispensing, n (%)	2225 (15.7)	286 (23.6)	1728 (14.2)
No. dispensings among people with ≥1 dispensing, median (IQR)	10 (6-12)	11 (6-12)	10 (6-12)
**Antibiotics**			
≥1 dispensing, n (%)	6219 (43.8)	657 (54.3)	5037 (41.5)
No. dispensings among people with ≥1 dispensing, median (IQR)	2 (1-4)	3 (1-5)	2 (1-3)
**Gastro-oesophageal reflux disease (GORD)**			
≥1 dispensing, n (%)	2703 (19.0)	389 (32.1)	1966 (16.2)
No. dispensings among people with ≥1 dispensing, median (IQR)	6 (2-12)	9 (3-12)	5 (1-11)

*excludes n=860 where involvement of medicines in poisoning could not be ruled out

Supplementary Table 3. Initiation rates of medicines by cause of death in the 3 months prior to death by suicide (2013-19). Class initiation refers to initiation of any medicine within the class, while medicine initiation refers to initiation of an individual medicine.

			Cause of death*			
	Overall (n=14207)		Medicine poisoning (n=1210)		Other causes (n=12137)	
**Class-level initiation among treatment-naïve**	**No. initiators / No. treatment-naïve**	**%**	**No. initiators / No. treatment-naïve**	**%**	**No. initiators / No. treatment-naïve**	**%**
**Nervous system medicines**						
Antidepressants	1054 / 8623	12.2	61 / 516	11.8	961 / 7894	12.2
Benzodiazepines	1035 / 10547	9.8	100 / 688	14.5	867 / 9487	9.1
Antipsychotics	429 / 11947	3.6	37 / 937	4.0	365 / 10426	3.5
Opioids	538 / 10901	4.9	56 / 736	7.6	436 / 9733	4.5
**Non-nervous system medicines**						
ACEI/ARBs	100 / 12082	0.8	14 / 938	1.5	78 / 10487	0.7
GORD medicines	278 / 11782	2.4	32 / 853	3.8	225 / 10396	2.2
Antibiotics	819 / 8807	9.3	71 / 624	11.4	694 / 7794	8.9
**Medicine-level initiation among full population**	**No. initiators / Total population**	**%**	**No. initiators / Total population**	**%**	**No. initiators / Total population**	**%**
**Nervous system medicines**						
Antidepressants	1823 / 14207	12.8	139 / 1210	11.5	1542 / 12137	12.7
Benzodiazepines	1355 / 14207	9.5	142 / 1210	11.7	1094 / 12137	9.0
Antipsychotics	662 / 14207	4.7	68 / 1210	5.6	542 / 12137	4.5
Opioids	854 / 14207	6.0	116 / 1210	9.6	635 / 12137	5.2
**Non-nervous system medicines**						
ACEI/ARBs	201 / 14207	1.4	25 / 1210	2.1	154 / 12137	1.3
GORD medicines	395 / 14207	2.8	48 / 1210	4.0	305 / 12137	2.5
Antibiotics	1757 / 14207	12.4	201 / 1210	16.6	1398 / 12137	11.5

*excludes n = 860 where involvement of medicines in poisoning could not be definitively determined

Supplementary Table 4. Weekly change in slope (dispensing rate per 1000 people per week) and timing of change in slope for dispensing of medicine classes stratified by cause of death (2013-19).

	Weekly dispensings per 1000 people, median (IQR)	Slope in first segment (95% CI)	Change point (weeks prior to death)	Slope in second segment (95% CI)
**All nervous system medicines**				
Medicine poisoning	460 (439-484)	1.57 (1.07 to 2.06)	--	--
Other causes	166 (159-183)	0.34 (0.20 to 0.47)	18 weeks	3.01 (2.54 to 3.47)
**Non-nervous system medicines**				
Medicine poisoning	423 (400-444)	0.18 (-0.34 to 0.70)	--	--
Other causes	184 (178-191)	0.34 (0.20 to 0.47)	4 weeks	-3.58 (-9.50 to 2.23)
**Antidepressants**				
Medicine poisoning	129 (123-138)	0.44 (0.23 to 0.65)	--	--
Other causes	61 (58-68)	0.18 (0.09 to 0.28)	14 weeks	1.68 (1.20 to 2.15)
**Benzodiazepines**				
Medicine poisoning	87 (82-95)	0.18 (-0.01 to 0.37)	8 weeks	4.12 (0.98 to 7.26)
Other causes	31 (29-34)	0.18 (0.14 to 0.22)	8 weeks	2.41 (1.90 to 2.91)
**Antipsychotics**				
Medicine poisoning	50 (43-57)	0.20 (0.04 to 0.37)	--	--
Other causes	24 (23-26)	0.05 (-0.02 to 0.11)	23 weeks	0.28 (0.19 to 0.37)
**Opioids**				
Medicine poisoning	124 (116-135)	0.39 (0.19 to 0.59)	--	--
Other causes	30 (28-33)	0.09 (0.007 to 0.17)	22 weeks	0.32 (0.19 to 0.44)
**Antibiotics**				
Medicine poisoning	46 (40-52)	0.18 (-0.05 to 0.41)	16 weeks	-0.64 (-1.51 to 0.24)
Other causes	23 (22-24)	0.01 (-0.01 to 0.04)	–	--
**Gastro-oesophageal reflux medicines**				
Medicine poisoning	50 (48-56)	0.01 (-0.01 to 0.13)	–	--
Other causes	20 (19-21)	0.04 (0.01 to 0.07)	–	--
**ACEI/ARBs**				
Medicine poisoning	43 (39-46)	0.05 (-0.08 to 0.18)	6 weeks	-2.40 (-6.20 to 1.41)
Other causes	25 (24-26)	-0.02 (-0.05 to 0.01)	5 weeks	-0.91 (-1.89 to 0.08)

Supplementary Table 5. Weekly change in slope (dispensing rate per 1000 people per week) and timing of change in slope stratified by cause of death, among people where each medicine class was detected by toxicology at death (2013-19).

	Weekly dispensings per 1000 people, median (IQR)	Slope in first segment (95% CI)	Change point (weeks prior to death)	Slope in second segment (95% CI)
**Antidepressants**				
Medicine poisoning	220 (202-240)	1.07 (0.66 to 1.49)	5 weeks	14.94 (-2.93 to 32.81)
Not medicine poisoning	159 (147-182)	0.82 (0.55 to 1.09)	14 weeks	6.86 (5.46 to 8.26)
**Benzodiazepines**				
Medicine poisoning	147 (137-165)	0.12 (-0.45 to 0.70)	18 weeks	3.16 (1.66 to 4.66)
Not medicine poisoning	94 (90-109)	0.62 (0.46 to 0.78)	8 weeks	11.83 (9.18 to 14.48)
**Antipsychotics**				
Medicine poisoning	176 (150-209)	0.99 (0.28 to 1.70)	--	
Not medicine poisoning	145 (135-161)	0.32 (-0.13 to 0.77)	19 weeks	3.72 (2.69 to 4.75)
**Opioids**				
Medicine poisoning	229 (213-254)	1.01 (0.60 to 1.43)	--	
Not medicine poisoning	130 (121-145)	0.63 (0.33 to 0.94)	17 weeks	3.38 (2.36 to 4.41)

Supplementary Table 6. Weekly change in slope (initiation rate per 1000 treatment-naïve people per two week period) and timing of change in slope stratified by cause of death (2013-19).

	People initiating per 1000 treatment-naïve per two-week period, median (IQR)	Slope (change per fortnight) (95% CI)	Change point (weeks prior to death)	Slope in second segment (95% CI)
**Antidepressants**				
Medicine poisoning	16 (12-19)	0.46 (-0.29 to 1.21)	--	
Other causes	11 (10-17)	0.34 (-0.36 to 1.04)	10 fortnights	3.54 (2.11 to 4.98)
**Benzodiazepines**				
Medicine poisoning	15 (13-20)	-1.20 (-2.58 to 0.18)	14 fortnights	3.40 (2.30 to 4.49)
Other causes	9 (8-13)	-0.30 (-0.88 to 0.27)	14 fortnights	2.19 (1.46 to 2.91)

Supplementary Figure 1. Medicine dispensing by week prior to death, stratified by cause of death, age and sex (2013-19). Rates calculated from counts <6 are redacted from the plots.

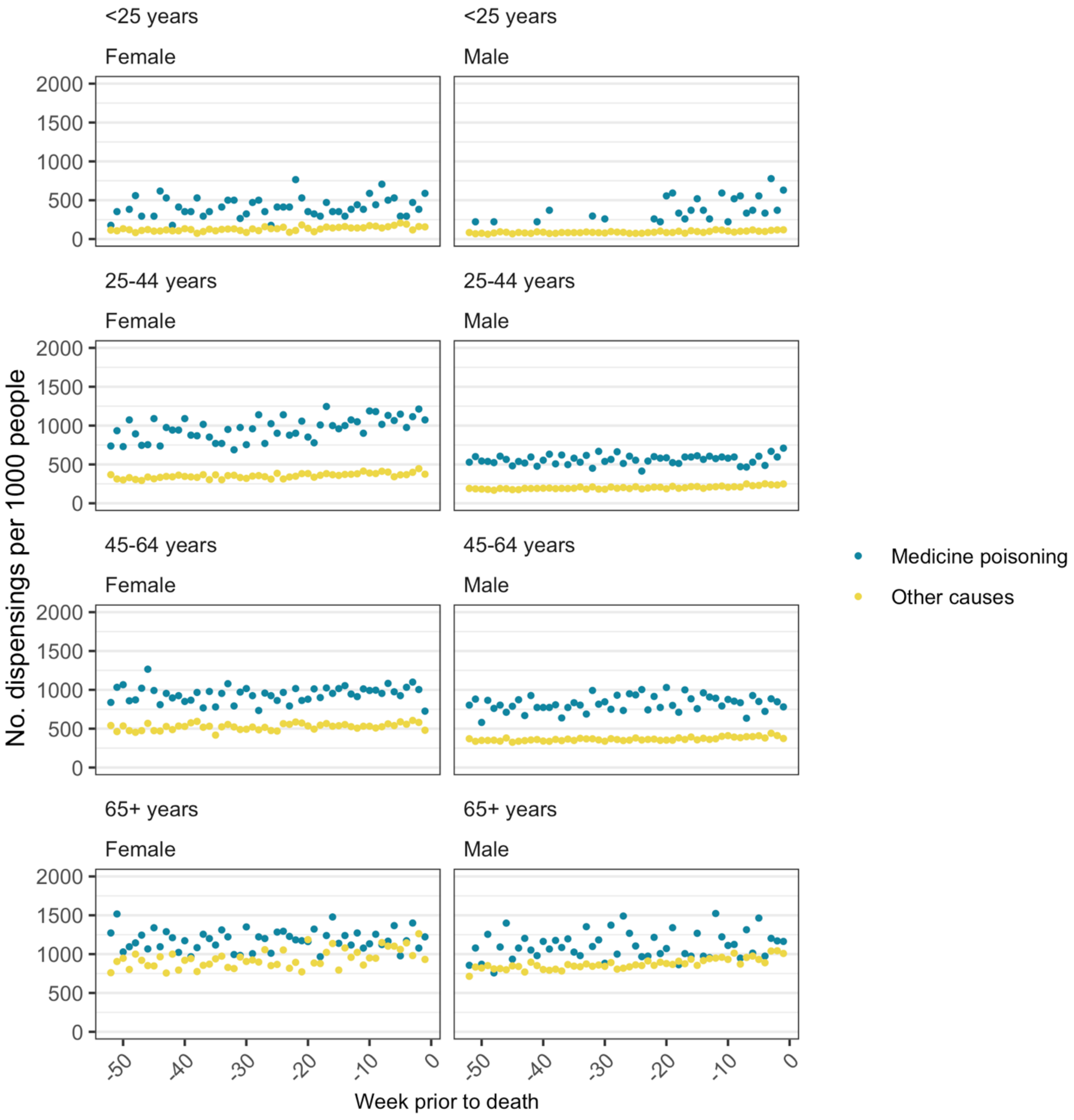

Supplementary Figure 2. Non-nervous system medicine dispensing by week prior to death, stratified by cause of death (2013-19). Points represent observed values, the solid line represents predicted values, shaded area represents 95% confidence interval for predicted values.

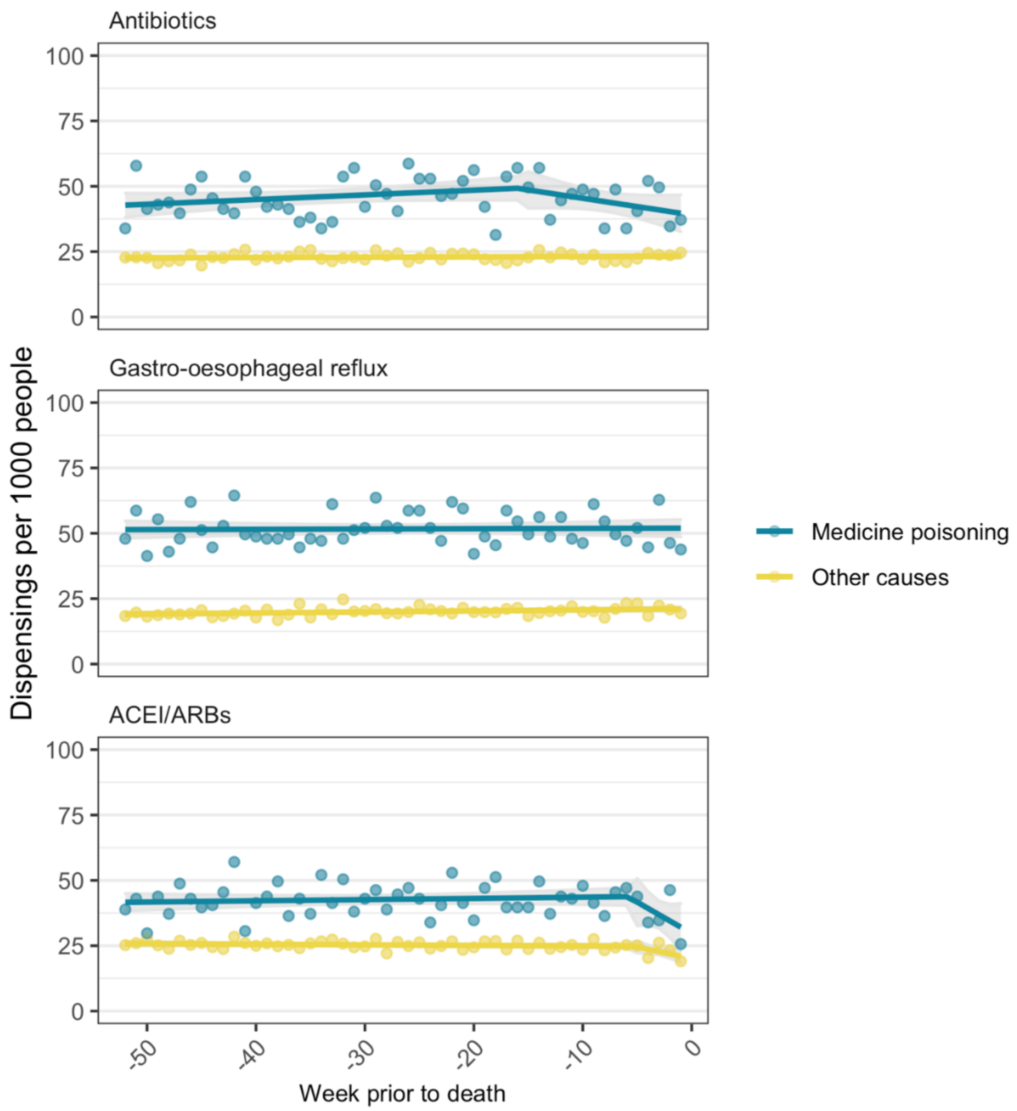

Supplementary Figure 3. Medicine dispensing by week prior to death among people where each medicine class was detected by toxicology at death, stratified by cause of death (2013-19). Points represent observed values, the solid line represents predicted values, shaded area represents 95% confidence interval for predicted values.

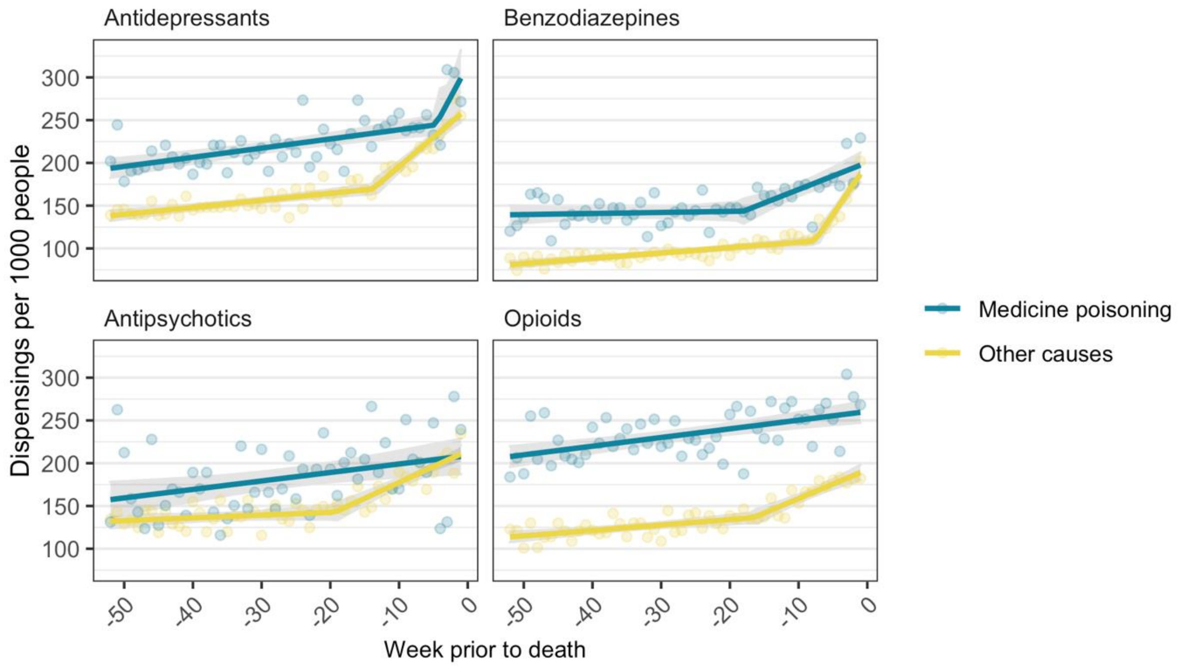

*opioids detected in toxicology includes both prescribed opioids and illicit opiates
